# Evaluation of Hypoglycemic and Genotoxic Effect of Polyphenolic Bark Extract from* Quercus sideroxyla*

**DOI:** 10.1155/2016/4032618

**Published:** 2016-10-27

**Authors:** Marcela Soto-García, Martha Rosales-Castro, Gerardo N. Escalona-Cardoso, Norma Paniagua-Castro

**Affiliations:** ^1^CIIDIR-Unidad Durango, Instituto Politécnico Nacional, Sigma 119 Fraccionamiento 20 de Noviembre, 34220 Durango, DGO, Mexico; ^2^Escuela Nacional de Ciencias Biológicas, Instituto Politécnico Nacional, Wilfrido Massieu s/n, Esquina Manuel L. Stampa, Colonia Unidad Profesional Adolfo López Mateos, Delegación Gustavo A. Madero, 07738 Ciudad de México, Mexico

## Abstract

*Quercus sideroxyla* is a wood species whose bark has phenolic compound and should be considered to be bioactive; the hypoglycemic and genotoxic properties of* Q*.* sideroxyla* bark were evaluated in this study. Total phenolic compound was determined in crude extract (CE) and organic extract (OE). The OE has the highest amount of phenols (724.1 ± 12.0 GAE/g). Besides, both CE and OE demonstrated effect over the inhibition of *α*-amylase* in vitro*. Hypoglycemic activity was assessed by glucose tolerance curve and the area under curve (UAC); OE showed the highest hypoglycemic activity. In addition, diabetes was induced by streptozotocin (65 mg/kg) and the extracts (50 mg/kg) were administered for 10 days; OE showed hypoglycemic effect compared with diabetic control and decreased hepatic lipid peroxidation. Acute toxicity and genotoxicity were evaluated in CE; results of acute toxicity did not show any mortality. Besides, the comet assay showed that CE at a dose of 100 mg/kg did not show any genotoxic effect when evaluated at 24 h, whereas it induced slight damage at 200 mg/kg, with the formation of type 1 comets.

## 1. Introduction

*Quercus* species have been used in Mexican traditional medicine [1]; specifically* Q. sideroxyla* has antioxidant compounds present in leaves [2] which decrease the levels of inflammatory markers such as COX-2 and IL-8 by modulating the expression of NF-kB [3]. Ten different polyphenols had been reported in the bark of this species [4], which are bioactive phytochemicals [5], found in green tea and found to possess hypoglycemic properties [6, 7].

Polyphenols are the most abundant dietary antioxidants and are common constituents of many plant food sources, including fruits, vegetables, seeds, chocolate, wine, coffee, and tea; thus they have acquired significant interest [8].

Recent studies have shown promising results for these compounds in various pathological conditions such as diabetes, cancer, atherosclerosis, cardiovascular, and neurological disorders [9–11].

The efficacy of polyphenols on carbohydrate metabolism and glucose homeostasis has been investigated* in vitro*, in animal models and clinical trials [12]. The polyphenols regulate the postprandial hyperglycemia through inhibiting carbohydrate-hydrolyzing enzymes such as alpha-amylase and alpha-glucosidase [13].

The origin of these bioactive compounds makes them safe for human consumption; however, some investigations reported toxic effects caused by plants [14–16]. Therefore, it is important to carry out toxicological and genotoxic assays [17], to assess the risk/benefit of its therapeutic use in humans.

Therefore, the aim of this research was to evaluate the hypoglycemic and genotoxic properties of polyphenolic extracts from* Q. sideroxyla* bark in a diabetic murine model.

## 2. Materials and Methods

### 2.1. Chemicals

Ethanol was purchased from Merck KGaA (Darmstadt, Germany), and gallic acid, Folin-Ciocalteu, streptozotocin, *α*-amylase enzyme, and acarbose were acquired from Sigma Chemical Co. All reagents were of analytical grade.

### 2.2. Plant Material

The bark was collected at Pueblo Nuevo, Durango, México. The samples were identified and botanical specimens were deposited at the Herbarium CIIDIR-Instituto Politécnico Nacional, Durango, with voucher numbers 42842, 428443, and 42844. The bark was mixed, to make a unique sample, and then it was dried at room temperature (24°C), milled (mesh 40), and finally stored in paper bags under refrigeration until further use.

### 2.3. Extract Preparation and Purification

The bark powder (10 g) was twice soaked with 50% ethanol (ethanol/water 50 : 50 v/v), (2 × 300 mL) at room temperature for 24 h, with stirring, followed by filtration through Whatman no. 1 filter paper. The extracts were combined, filtered, and then evaporated under vacuum at 40°C until ethanol was removed. A portion of the remaining aqueous extract was taken to dryness and identified as crude extract (CE), while another portion was subjected to liquid partition with ethyl acetate (3 × 100 mL). The organic phase was evaporated to dryness under vacuum at 40°C and identified as the organic extract (OE).

### 2.4. Evaluation of the Phenolic Compounds

The concentration of the total phenolic (TP) was determined by the Folin-Ciocalteu colorimetric method with slight modifications [11]. Measurements were carried out in duplicate and calculations based on a calibration curve were obtained with gallic acid. The total phenolic concentration was expressed as milligrams of gallic acid equivalents (GAE) per gram of dry extract.

### 2.5. *α*-Amylase Assay

The inhibition on alpha-amylase enzyme was determined by Kwon et al. [18] method with some modifications. The IC_50_ values were determinate by linear regression analysis using four different concentrations for acarbose (1000, 2000, 4000, and 5000 *μ*g/mL), CE, and OE (500, 1000, 1500, and 2000 *μ*g/mL) in triplicate. Acarbose was used like drug reference and control, respectively. The reaction was stopped with 1.0 mL of color reagent (contains the mixture of dinitrosalicylic acid 96 M and sodium potassium tartrate 0.005 M); results show mean of the data.

### 2.6. Animals

Healthy Wistar rats were obtained from the Biorepository of the National School of Biological Sciences (National Polytechnic Institute, México) and ICR mice were obtained from PROPECIA S.A., México. All experiments were approved by the Laboratory Animal Care Committee of the National School of Biological Sciences (National Polytechnic Institute, México) and were conducted in compliance with the Mexican Official Standard (NOM—062-200-1999) technical specifications for the production, care, and use of laboratory animals. The animals were group-housed in polycarbonate cages in a controlled environment at a constant temperature (21 ± 2°C) with 12 h light/dark cycles and access to food and fresh water* ad libitum*.

### 2.7. Evaluation of Hypoglycemic Properties in Healthy Rats

The hypoglycemic activity was evaluated using a glucose tolerance test in male Wistar rats weighing 300 ± 10 g. The animals were fasted for 12 h before testing and basal blood samples were obtained from the tip of the tail. Three groups of 5 animals each received CE by gavage. One group received 50 mg/kg body weight (CE50), another group 100 mg/kg body weight (CE100), and the last group received 200 mg/kg body weight crude extract (CE200); in the same way two groups of animals (*n* = 5/group) received OE at doses of 25 (OE25) and 50 mg/kg body weight (OE50) at time zero. Doses were selected according to the content of phenols. After fifteen minutes, all animals received an oral dose of glucose (3 g/kg body weight) using a 35% solution. An additional control group of 6 animals was given only glucose. At 0, 30, 60, 90, and 120 min after glucose administration, blood glucose was measured using glucose test strips in glucometer (Accu-Check Performa with Softclix, Roche) and then the area under the curve was calculated to estimate the glucose tolerance.

### 2.8. Hypoglycemic Effect in Diabetic Rats

The hypoglycemic effect of the extracts was tested in male Wistar rats (5 animals/dose of extract) with previously induced type I diabetes by intraperitoneal streptozotocin injection (STZ, 65 mg/kg of body weight, in citrate buffer, pH 4.4). Diabetes was confirmed by measuring the glucose levels in blood samples obtained of the tip of the tail, 24 hours after. Five days later, animals received 50 mg/kg body weight CE and OE of* Q. sideroxyla* for 10 days; doses were selected by their hypoglycemic action that showed before section. Blood glucose was determined with glucose test strips (Accu-Check Performa with Softclix, Roche) and compared with a nondiabetic control group and untreated diabetic control group. After the animals were euthanized, fasted liver samples were taken for lipid peroxidation assays.

### 2.9. Hepatic Lipid Peroxidation Assay

Oxidative stress levels were evaluated by the concentration of malondialdehyde (MDA) in the liver according to Rivera-Ramírez et al. [19]. The MDA concentration was calculated using the molar extinction coefficient of 1.56 × 10^−5^ M^−1^ cm^−1^. The results were expressed in mmol MDA/g tissue.

### 2.10. Acute Toxicity Study in Healthy Mice

Evaluation of the toxicity of* Q. sideroxyla* bark extracts was made according to Lorke [20]. Four groups containing healthy male ICR mice (*n* = 3/group) received CE, at doses of 1000, 2000, 3000, and 5000 mg/kg body weight. The toxicological effects were expressed in terms of mortality expressed as LD_50_. Special attention was directed to the observations of convulsions, diarrhea, lethargy, and piloerection. The number of animals dying during a period was recorded.

### 2.11. Genotoxic Assay

Thirty-six male mice weighing 25–30 g were divided into six experimental groups of six animals each. The crude extracts were suspended in 1% Tween-80 aqueous solution and it was administered by gavage at doses of 100 and 200 mg/kg body weight. The negative group received 1% Tween-80 aqueous solution, and the positive control group received an intraperitoneal injection of cyclophosphamide (CPA) at 50 mg/kg body weight. The evaluation of DNA damage was done by comet assay according to Almonte-Flores et al. [21] at 4 and 24 hours, where it was evaluated by examining 100 randomly selected cells (50 cells per coded slide) per animal. These cells were scored visually according to tail size and grouped into the following four classes: class 0, no tail; class 1, tail shorter than the diameter of the head (nucleus); class 2, tail length 1-2 times the diameter of the head; and class 3, tail length more than twice the diameter of the head. The total comet score was calculated by the following equation:

(1) Total comet =% of cells in class 0∗0+% of cells in class 1∗1+% of cells in class 2∗2+% cell in class 3∗3.

### 2.12. Statistical Analyses

The results are expressed as the mean and the standard deviation. One-way analyses of variance (ANOVA) were performed followed by multiple comparisons of the means with the Fisher-LSD test at a significance level of *α* < 0.5. The statistical analysis software package (Statistica 7) was used for these analyses.

## 3. Results

The evaluation of phenols compounds of CE and OE from* Q. sideroxyla* bark revealed that OE has a significant higher concentration of these metabolites than CE (*p* ≤ 0.05) (Table 1). In the same table, the antidiabetic activity of CE and OE extracts was demonstrated through their higher inhibition (IC_50_ 1979.3 and 1703.3, resp.) of *α*-amylase activity compared to acarbose (IC_50_ 4030.1).

**Figure 1:**
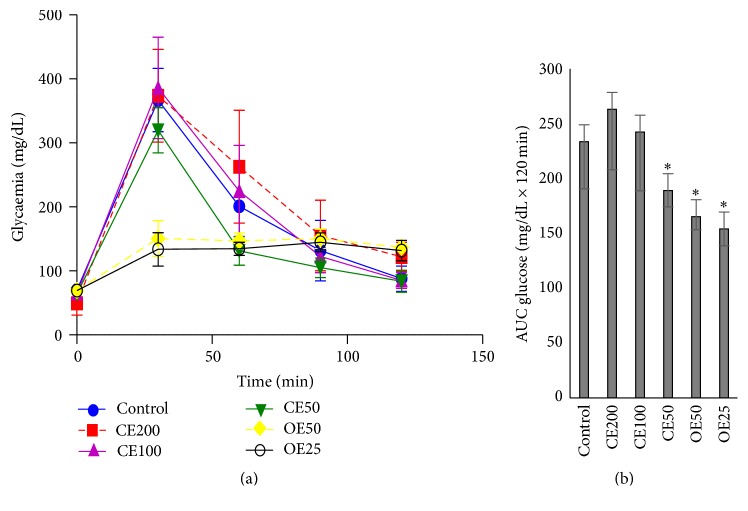
Glucose tolerance test in rats (a). Area under the glucose curve (b). ^*∗*^ *p* ≤ 0.05, significant difference compared with the control group. CE: crude extract at doses 200, 100, and 50 mg/kg; OE: organic extract at doses 50 and 25 mg/kg.

In regard to the glucose tolerance test in rats, a similar behavior is observed by the organic extracts (Figure 1(a)). The extracts OE50, OE25, and CE50 significantly decreased blood glucose levels (165.3 ± 15.4 mg/dL min; 154.1 ± 11.8 mg/dL min; and 189.1 ± 14.9 mg/dL min, resp.) compared with the control group (233.7 ± 43.0 g/L min) (Figure 1(b)). Both OE50 and OE25 showed a high antihyperglycemic activity, and the former was chosen to be compared with CE50; this is in order to observe the action of all compounds in extract, as well as the effect of the extract with the selectivity of components, using a solvent semipurification.

The effect of repeated oral administration of CE50 and OE50 on blood glucose levels in STZ-diabetic rats is present in Table 2. The OE50 showed a lowering effect on glucose in diabetic rats, compared to diabetic control (*p* ≤ 0.05), and was more effective in reducing blood glucose than the CE50 (*p* ≤ 0.05).

**Figure 2:**
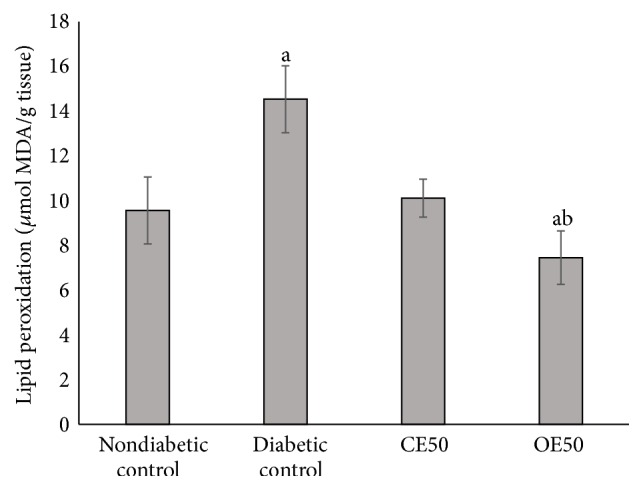
Hepatic lipid peroxidation assay in diabetic rats treated with CE50 and OE50 extracts during 10 days. a: significant difference versus control nondiabetic group; b: versus diabetic control group (*p* ≤ 0.05), Fisher-LSD test. CE: crude extract 50 mg/kg; OE: organic extract 50 mg/kg.

The evaluation of hepatic lipid peroxidation revealed that the concentration of malondialdehyde (MDA) was significantly higher in diabetic control (Figure 2). On the other hand, the diabetic rats that received CE50 and OE50 have reduced the concentration of MDA, in the case of OE50 even to lower level than the healthy control (*p* ≤ 0.05).

The administration in healthy rats of 5000 mg/kg body weight of CE produced no mortality, so LD_50_ ≥ 5000 mg/kg. The animals did not manifest any sign of convulsions, diarrhea, lethargy, or piloerection.

**Figure 3:**
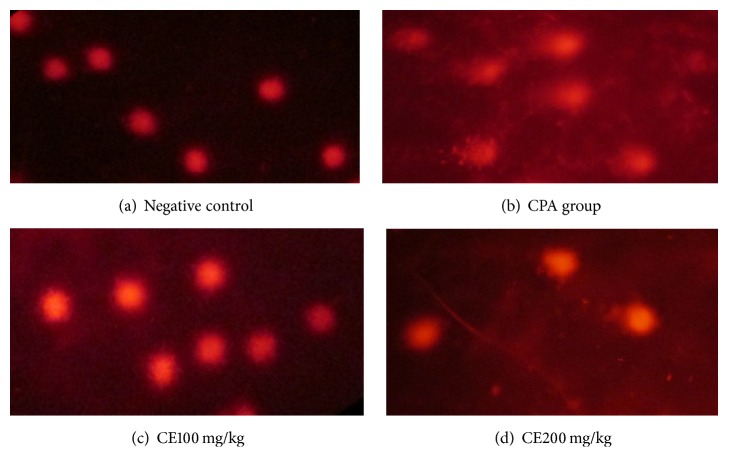
Comet assay images in cell blood of mice (400x). (a) Comet type 0, (b) comet type 3, (c) comet type 1, and (d) comet types 1 and 2.

The results of the genotoxicity assay are shown in Table 3 and Figure 3. The test showed that the CPA group has the highest levels of damage, according to comet class detected (comet classes 2 and 3), while the negative control presented a predominance of comet zero class.

In regard to genotoxicity, the evaluation of CE100 after 24 h revealed no significant differences compared to the negative control (*p* ≤ 0.05); nevertheless CE200 after 4 h and CE200 after 24 h showed light damage with the formation of comets of type 1 and showed significant differences with the negative control (*p* ≤ 0.05).

## 4. Discussion

Total phenolic content in extracts of* Q. sideroxyla* bark (CE and OE) differed and is significantly highest in OE (724.1 ± 12 GAE/g), which suggests that the solvent has a concentrating effect of these metabolites. Phenolic compounds as gallic acid, catechin, epicatechin, gallocatechin, dimeric, and dimeric procyanidins have been identified in* Q. sideroxyla* bark [4]. Those polyphenols are bioactive phytochemicals with antioxidant properties. Antioxidants are important in preventing pathologies like diabetes, so low levels of plasma antioxidants imply a risk factor for the development of the disease [22].

This research demonstrates the inhibitory activity of the CE and OE in *α*-amylase enzyme. We suggest that the effect is due to the synergy of the phenolic compounds present in the extracts, because the inhibitory activity of acarbose on the enzyme was lower. Moreover, antioxidants have been reported because of their capacity to contribute to the prevention of type 2 diabetes through their anti-inflammatory properties [23]; therefore both mechanisms antioxidant effect and *α*-amylase inhibition could be summarized to produce antihyperglycemic effect.

Inhibiting glucose uptake in the intestines may help to control the blood glucose, since it is known that the *α*-amylase is one of the main products of secretion from glands in the pancreas and salivary glands, which play an important role in digestion, by participating in the intestinal absorption; it is believed that inhibition of these enzymes can significantly reduce the postprandial increase in blood glucose level, which is important in the treatment of diabetes [24, 25]. For this reason, the bark of* Q. sideroxyla* could be used as an effective treatment for the management of postprandial hyperglycemia and to limit complications of diabetes.

The antihyperglycemic activity of CE (50 mg/kg b.w.) and OE (50 mg/kg/b.w.) in diabetic rats shows significant differences in respect to the diabetic group. Besides that OE can eliminate hyperglycemic peaks so as to maintain the glucose levels to more stables levels. Despite the fact that CE and OE showed a hypoglycemic effect at the beginning of the treatment, this was lost in the case of CE as the treatment continues. Thus, it is possible that inhibition of *α*-amylase activity by phenolic compounds and the antioxidant effect by compounds like catechin are mechanisms associated with the pharmacologic properties from bioactive compounds of* Q. sideroxyla* bark. The higher hypoglycemic effect shown by OE could be explained by the higher concentration of phenols compared to CE. It is necessary to do more experiments to demonstrate that CE and OE extracts inhibit carbohydrate absorption in gut.

Several studies have demonstrated that the administration of plant extracts containing phenols decreased glucose levels by the presence of flavonoids such as quercetin and catechin which promote a major hepatic glycogen storage [26, 27]. Other possible mechanisms involve the activity of procyanidin that inhibits the activity of carbohydrate-hydrolyzing enzymes (found in results of *α*-amylase assay), epicatechin that induces pancreatic *β* cell regeneration, and catechin and phenolics acids that inhibit intestinal glucose absorption mediated by GLUT 2 and SGLT1 [28, 29].

In diabetes, tissue damage is mediated by free radicals by attacking membranes through lipid peroxidation. The concentration of MDA reflects the degree of lipid peroxidation, and the increase of MDA production plays a key factor in the progression of diabetic pancreas damage [22, 30]. Therefore, a decrease in the peroxidation of fatty acids and improved antioxidant status could contribute to prevention of diabetic complications. In our study, the MDA levels were higher in the diabetic control, in comparison with the diabetic rats treated with CE and OE which decreased the lipid peroxidation (*p* ≤ 0.05). This can be explained by the antioxidant effect of polyphenols present in both extracts, which has been observed in other studies from plants [31, 32].

The toxicity results showed that CE100 was not considered a genotoxic agent in this study; however a low level of genotoxicity was observed with CE200. Some studies showed a dose-dependent relationship and even talk about the possible activation of different cellular pathways depending on the tested dose [21, 33]. It has been shown that flavonoids have effects against DNA damage induced by various genotoxic agents, that is, principally by the ability to protect against ROS produced and by the modulation of enzymes responsible for bioactivation and detoxification of genotoxic agents. A relationship has been reported between structure and activity from flavonoids in the protection of genetic material [34].

This research provides the first report on hypoglycemic and genotoxic effect of the extract from* Q. sideroxyla* bark. Polyphenols may explain this activity; however further studies focusing on the mechanism of action are required; in addition the toxicological analysis of OE is necessary.

## 5. Conclusion

We conclude that extracts of* Quercus sideroxyla* bark at doses of 50 mg/kg may be effective in reducing postprandial hyperglycemia. However, CE extract at doses of 200 mg/kg produces a low level DNA damage. It is necessary to do more studies to evaluate its potential usefulness as an adjuvant for diabetes treatment.

## Figures and Tables

**Table 1:** Concentration of phenolic compound and IC_50_ of *α*-amylase of CE of *Q. sideroxyla* bark extracts.

Extract/standard	Total phenolic compound (GAE/g)	IC_50_ (*μ*g/mL)
CE	551.0 ± 8.1^a^	1979.3 ± 1.2^a^
OE	724.1 ± 12.0^b^	1703.3 ± 21.6^b^
Acarbose	—	4030.1 ± 12.2^c^

Each value represents the mean of *n* = 4  ± standard deviation. Different letters between groups in each column indicate a significant difference (*p* ≤ 0.05) assessed using the Fisher-LSD test.

**Table 2:** Blood glucose levels during the study period in Wistar rats.

Group	Blood glucose (mg/dL)			
	Day 1	Day 3	Day 6	Day 10
Control	89.6 ± 5.1	92 ± 8.6	100.4 ± 7.5	91 ± 22.9
Diabetic	419.6 ± 49.6^a^	442.2 ± 36.7^a^	500 ± 3.1^a^	500 ± 0^a^
Diabetic + CE50	462 ± 33.9^a^	397.2 ± 61.2^a^	465.5 ± 33.3^a^	500 ± 0^a^
Diabetic + OE50	397 ± 10.4^ab^	337.8 ± 34.6^ab^	372.6 ± 7.5^ab^	414.2 ± 62.5^ab^

Data are expressed as mean ± standard deviation; *n* = 5. a: significant difference versus control group; b: versus diabetic group (*p* ≤ 0.05) in Fisher-LSD test. CE50: crude extract at dose 50 mg/kg; OE50: organic extract at dose 50 mg/kg.

**Table 3:** DNA damage according to the comet assay for the assessment of the genotoxicity of polyphenolic extract from *Q. sideroxyla* bark.

Treatment		Comet class			
	Total	0	1	2	3
Blood sample (4 h)					
Negative control	8 ± 4.8	92.5 ± 4.1	7 ± 3.5	0.5 ± 0.8	0 ± 0
CPA (50 mg/kg)	67.3 ± 6.9^a^	66 ± 3.1^a^	11.8 ± 3.3^a^	11 ± 2.4^a^	11.1 ± 1.0^a^
CE100	16.8 ± 6.3^ab^	85.8 ± 4.9^b^	12.1 ± 3.5^a^	1.33 ± 0.8^b^	0.66 ± 0.8
CE200	39.5 ± 6.2^abc^	69.8 ± 5.1^a^	22.5 ± 6.0^ab^	6 ± 2.09^abc^	1.6 ± 0.8^b^
Blood sample (24 h)					
Negative control	18.5 ± 6.9	88.6 ± 3.1	7.6 ± 3.3	3.1 ± 2.4	1.5 ± 1.04
CPA (50 mg/kg)	140.3 ± 21.2^a^	37.5 ± 5.5^a^	11 ± 6.2^a^	25.2 ± 8.4^a^	26.3 ± 6.3^a^
CE100	21.3 ± 6.1^b^	85.5 ± 4.0^b^	9.1 ± 0.7^a^	3.3 ± 1.6^b^	1.8 ± 1.4^b^
CE200	60 ± 14.3^ab^	55.3 ± 5.9^ab^	33.3 ± 3.4^ab^	7.3 ± 4.0^b^	4 ± 2.6^b^

Each value represents the mean of *n* = 6 observations ± standard deviation. a: significant difference versus negative control group, b: versus CPA 50 mg/kg, and c: versus CE100; (*p* ≤ 0.05) LSD, comparing the treatments separately at 4 and 24 hours.

CPA: group treated with cyclophosphamide; CE100: group treated with crude extract at dose of 100 mg/kg; CE200: group treated with crude extract at dose of 200 mg/kg.

## References

[1] Luna-José A. D. L., Montalvo-Espinosa L., Rendón-Aguilar B. E. A. T. R. I. Z. (2003). Los usos no leñosos de los encinos en México. *Boletín de la Sociedad Botánica de México*.

[2] Rivas-Arreola M. J., Rocha-Guzmán N. E., Gallegos-Infante J. A. (2010). Antioxidant activity of oak (*Quercus*) leaves infusions against free radicals and their cardioprotective potential. *Pakistan Journal of Biological Sciences*.

[3] Moreno-Jimenez M. R., Trujillo-Esquivel F., Gallegos-Corona M. A. (2015). Antioxidant, anti-inflammatory and anticarcinogenic activities of edible red oak (*Quercus* spp.) infusions in rat colon carcinogenesis induced by 1,2-dimethylhydrazine. *Food and Chemical Toxicology*.

[4] Rosales-Castro M., González-Laredo R. F., Rocha-Guzmán N. E., Gallegos-Infante J. A., José Rivas-Arreola M. J., Karchesy J. J. (2012). Antioxidant activity of fractions from *Quercus sideroxyla* bark and identification of proanthocyanidins by HPLC-DAD and HPLC-MS. *Holzforschung*.

[5] Pardos-Sevilla C., Mach N. (2014). Efectos del té verde sobre el riesgo de cáncer de mama. *Revista Española de Nutrición Humana y Dietética*.

[6] Yang X., Kong F. (2016). Effects of tea polyphenols and different teas on pancreatic *α*-amylase activity in vitro. *LWT—Food Science and Technology*.

[7] Xu Y., Zhang Z., Li L. (2013). Catechins play key role in green tea extract–induced postprandial hypoglycemic potential in vitro. *European Food Research and Technology*.

[8] Ly C., Yockell-Lelièvre J., Ferraro Z. M., Arnason J. T., Ferrier J., Gruslin A. (2015). The effects of dietary polyphenols on reproductive health and early development. *Human Reproduction Update*.

[9] Tomé-Carneiro J., Visioli F. (2016). Polyphenol-based nutraceuticals for the prevention and treatment of cardiovascular disease: review of human evidence. *Phytomedicine*.

[10] Xiao J., Högger P. (2014). Influence of diabetes on the pharmacokinetic behavior of natural polyphenols. *Current Drug Metabolism*.

[11] Pasinetti G. M., Wang J., Ho L., Zhao W., Dubner L. (2015). Roles of resveratrol and other grape-derived polyphenols in Alzheimer's disease prevention and treatment. *Biochimica et Biophysica Acta (BBA)—Molecular Basis of Disease*.

[12] Bahadoran Z., Mirmiran P., Azizi F. (2013). Dietary polyphenols as potential nutraceuticals in management of diabetes: a review. *Journal of Diabetes and Metabolic Disorders*.

[13] Olubomehin O. O., Abo K. A., Ajaiyeoba E. O. (2013). Alpha-amylase inhibitory activity of two *Anthocleista* species and *in vivo* rat model anti-diabetic activities of *Anthocleista djalonensis* extracts and fractions. *Journal of Ethnopharmacology*.

[14] Navarro E. S., del Carmen Rodríguez M., Patterson D. C., Arcia I. R. (2014). Intoxicación por tóxico vegetal de la planta Ackee. Reporte de un caso. *Mediciego*.

[15] Torrico F., Ramos k., Morales A. (2014). Evaluación de la toxicidad aguda, actividad analgésica e hipoglicemiante del extracto acuoso de Croton pungens en animales experimentales. *Ciencia*.

[16] Silvero-Isidre A., Morínigo-Guayuán S., Mongelós-Cardozo M., González-Ayala A., Figueredo-Thiel S. (2016). Toxicidad aguda de las hojas de *Xanthium spinosum* en ratones BALB/C. *Revista Peruana de Medicina Experimental y Salud Pública*.

[17] Penumetcha M., Santanam N. (2012). Nutraceuticals as ligands of PPAR*γ*. *PPAR Research*.

[18] Kwon Y.-I., Apostolidis E., Shetty K. (2008). In vitro studies of eggplant (*Solanum melongena*) phenolics as inhibitors of key enzymes relevant for type 2 diabetes and hypertension. *Bioresource Technology*.

[19] Rivera-Ramírez F., Escalona-Cardoso G. N., Garduño-Siciliano L., Galaviz-Hernández C., Paniagua-Castro N. (2011). Antiobesity and hypoglycaemic effects of aqueous extract of *Ibervillea sonorae* in mice fed a high-fat diet with fructose. *Journal of Biomedicine and Biotechnology*.

[20] Lorke D. (1983). A new approach to practical acute toxicity testing. *Archives of Toxicology*.

[21] Almonte-Flores D. C., Paniagua-Castro N., Escalona-Cardoso G., Rosales-Castro M. (2015). Pharmacological and genotoxic properties of polyphenolic extracts of *Cedrela odorata* L. and *Juglans regia* L. barks in rodents. *Evidence-Based Complementary and Alternative Medicine*.

[22] Huang D., Jiang Y., Chen W., Yao F., Huang G., Sun L. (2015). Evaluation of hypoglycemic effects of polyphenols and extracts from *Penthorum chinense*. *Journal of Ethnopharmacology*.

[23] Umeno A., Horie M., Murotomi K., Nakajima Y., Yoshida Y. (2016). Antioxidative and antidiabetic effects of natural polyphenols and isoflavones. *Molecules*.

[24] Giordani M. A., Collicchio T. C. M., Ascêncio S. D. (2015). Hydroethanolic extract of the inner stem bark of *Cedrela odorata* has low toxicity and reduces hyperglycemia induced by an overload of sucrose and glucose. *Journal of Ethnopharmacology*.

[25] Lordan S., Smyth T. J., Soler-Vila A., Stanton C., Ross R. P. (2013). The *α*-amylase and *α*-glucosidase inhibitory effects of Irish seaweed extracts. *Food Chemistry*.

[26] Alam M. M., Meerza D., Naseem I. (2014). Protective effect of quercetin on hyperglycemia, oxidative stress and DNA damage in alloxan induced type 2 diabetic mice. *Life Sciences*.

[27] Vasconcelos C. F. B., Maranhão H. M. L., Batista T. M. (2011). Hypoglycaemic activity and molecular mechanisms of *Caesalpinia ferrea Martius* bark extract on streptozotocin-induced diabetes in Wistar rats. *Journal of Ethnopharmacology*.

[28] Parveen K., Khan M. R., Mujeeb M., Siddiqui W. A. (2010). Protective effects of Pycnogenol® on hyperglycemia-induced oxidative damage in the liver of type 2 diabetic rats. *Chemico-Biological Interactions*.

[29] Manzano S., Williamson G. (2010). Polyphenols and phenolic acids from strawberry and apple decrease glucose uptake and transport by human intestinal Caco-2 cells. *Molecular Nutrition & Food Research*.

[30] Ayala A., Muñoz M. F., Argüelles S. (2014). Lipid peroxidation: production, metabolism, and signaling mechanisms of malondialdehyde and 4-hydroxy-2-nonenal. *Oxidative Medicine and Cellular Longevity*.

[31] Moraes I. B., Manzan-Martins C., De Gouveia N. M. (2015). Polyploidy analysis and attenuation of oxidative stress in hepatic tissue of STZ-induced diabetic rats treated with an aqueous extract of *Vochysia rufa*. *Evidence-Based Complementary and Alternative Medicine*.

[32] Shetty B., Rao G., Banu N., Reddy S. (2016). Study of protective action of *Spondias pinnata* bark extract on rat liver and kidney against etoposide induced chemical stress. *Pharmacognosy Journal*.

[33] Alves A. B. C. R., dos Santos R. S., de Santana Calil S. (2014). Genotoxic assessment of *Rubus imperialis* (Rosaceae) extract *in vivo* and its potential chemoprevention against cyclophosphamide-induced DNA damage. *Journal of Ethnopharmacology*.

[34] Luca V. S., Miron A., Aprotosoaie A. C. (2016). The antigenotoxic potential of dietary flavonoids. *Phytochemistry Reviews*.

